# Identification of clinical trait-related small RNA biomarkers with weighted gene co-expression network analysis for personalized medicine in endocervical adenocarcinoma

**DOI:** 10.18632/aging.203543

**Published:** 2021-09-20

**Authors:** Zhiwen Shi, Xinyu Qu, Chenyan Guo, Lihong Zhang, Chuyue Peng, Zhu Xie, Keqin Hua, Junjun Qiu

**Affiliations:** 1 Obstetrics and Gynecology Hospital, Fudan University, Shanghai 200011, China; 2 Shanghai Key Laboratory of Female Reproductive Endocrine Related Diseases, Institute of Metabolism and Integrative Biology, Institutes of Biomedical Sciences, Fudan University, Shanghai 200433, China; 3 State Key Laboratory of Genetic Engineering, MOE Key Laboratory of Contemporary Anthropology, and Collaborative Innovation Center for Genetics and Development, School of Life Sciences, Fudan University, Shanghai 200438, China

**Keywords:** endocervical adenocarcinoma, small RNA sequencing, tDRs, WGCNA, biomarkers

## Abstract

Endocervical adenocarcinoma (EAC) is an aggressive type of endocervical cancer. At present, molecular research on EAC mainly focuses on the genome and mRNA transcriptome, the investigation of small RNAs in EAC has not been fully described. Here, we systematically explored small RNAs in 14 EAC patients with different subtypes using small RNA sequencing. MiRNAs and tRNA-derived RNAs (tDRs) accounted for the majority of mapped reads and the total number of miRNAs and tDRs maintained a relative balance. To explore the correlations between small RNAs expression and EAC with different clinical characteristics, we performed the weighted gene co-expression network analysis (WGCNA) and screened for hub small RNAs. From the key modules, we identified 9 small RNAs that were significantly related to clinical characteristics in EAC patients. Gene ontology and pathway analyses revealed that these molecules were involved in the pathogenesis of EAC. Our work provided new insights into EAC pathogenesis and successfully identified several small RNAs as candidate biomarkers for diagnosis and prognosis of EAC.

## INTRODUCTION

As the fourth most frequently diagnosed cancer and the fourth leading cause of cancer-related death, cervical cancer accounted for 570,000 new cases and 311,000 deaths in 2018 worldwide [1]. Endocervical adenocarcinoma (EAC) comprises 10–25% of cervical carcinoma, and has been increasing in incidence in recent years [2]. The management of EAC is currently based on International Federation of Gynecology and Obstetrics (FIGO) staging and National Comprehensive Cancer Network (NCCN) guidelines [3]. However, these systems aren’t yet comprehensive in clinical practice when applied to EAC. The FIGO staging usually takes into account the tumor size and extent of invasion, which are hard to measure in occult EAC. In addition, although EAC and squamous cervical carcinoma have different histological morphology, sites of origin and spreading patterns, they are staged and treated equally in accordance with NCCN guidelines [4]. Thus, these systems have limited reproducibility for staging EAC and may lead to inappropriate treatment decision making.

To overcome such deficiencies and provide clinically meaningful means of stratifying EAC, a novel Silva system was developed in 2013 based on the pattern of stromal invasion morphology [5]. Pattern A is characterized by well-demarcated glands with no destructive invasion or lymph-vascular space invasion (LVSI), pattern B represents localized destructive invasion, and pattern C demonstrates diffusely infiltrative glands. This new system was then validated in several subsequent studies and showed better performance in predicting nodal metastasis and prognosis [6–9]. Moreover, the treatment modality for each pattern has been proposed, and may help to develop a precise treatment-decision system based on different Silva patterns [10]. For Silva pattern A patients, adjuvant treatment and nodal sampling can be exempted with negative excision margins. The treatment of pattern B patients should be personalized based on the issue of lymphovascular invasion, while for pattern C patients, lymph node assessment and radical hysterectomy are required, if applicable, with additional adjuvant or preoperative therapy. Although this new Silva pattern can refine treatment strategies, it relies on postoperative histopathologic examinations in most cases and thus a preoperative biomarker to predict Silva patterns is urgently needed.

Biomarkers, such as proteins, metabolites and small molecules, are able to indicate specific processes, events or conditions [11, 12]. In the field of cancer, small molecules have been applied in numerous clinical scenarios to assist making diagnoses or evaluating prognosis [13]. Small RNAs (sRNAs) have been the focus of many researches in the last decades, among which are micro RNAs (miRNAs) with great potential for biomarker utility. Micro RNAs are 20-25nts in length and play an important role in multiple biological processes, mainly via post-transcriptional regulation of gene expression [14]. The potential of miRNAs in cancer diagnosis is increasingly recognized [15]. In addition to miRNAs, another type of sRNAs has emerged as critical regulators of gene expression - tRNA-derived small RNAs (tDRs) [16]. The tDRs have been identified to be involved in cell proliferation, apoptosis, and metastasis in various kinds of human carcinoma. These dysregulated tDRs interact with PIWI proteins to regulate gene expression in a sequence-specific manner [17]. Some of the newly identified tDRs have been considered as new biomarkers and therapeutic targets for the treatment of cancer. For example, tDRs have been exploited as diagnostic and prognostic biomarkers in chronic lymphocytic leukemia [18]. Taken together, these findings strongly suggest a functional role for miRNAs and tDRs in cancer progression. However, whether miRNAs and tDRs play a role in EAC is not well understood.

To fill this gap, we performed small RNA-sequencing on 14 EAC tissues of different Silva patterns with qPCR confirmation. In addition, we applied the weighted gene co-expression network analysis (WGCNA) to find the miRNAs and tDRs closely correlated with clinical traits. This study may provide a novel perspective into new biomarkers or therapeutic targets for EAC.

## RESULTS

### Characteristics of the study population

The clinicopathological characteristics of the 20 patients are listed in Table 1. The median follow-up period was 90 (18-162) months, during which 6 patients died and 7 patients experienced recurrences. Of the 20 patients, 14 patients were diagnosed with EAC, including 2 pattern A, 3 pattern B, 6 pattern C and 3 Gastric type. In terms of the clinical traits of EAC with different Silva patterns, patients with pattern C appear to have larger tumor sizes and deeper invasion than the other two patterns (Table 2).

**Table 1:** Baseline characteristics of the 20 patients.

**Characteristics**	**Number (n=20)**
**Age**	
Mean ± SD	46.7±9.6
Histology (%)	
SCC	2 (10)
AC	14 (70)
AS	2 (10)
NEC	2 (10)
**FIGO stage (%)**	
1B1	12 (60)
1B2	3 (15)
2A1	3 (15)
2A2	2 (10)
**Adjuvant treatment (%)**	
No	7 (35)
Yes	13 (65)
**HPV infect (%)**	
No	1 (5)
Yes	12 (60)
Unknown	7 (35)
**LN metastasis (%)**	
No	10 (50)
Yes	10 (50)
**Surgical margin (%)**	
No	20 (100)
Yes	0 (0)
**Parametrial invasion (%)**	
No	16 (80)
Yes	4 (20)
**Tumor size, cm (%)**	
≤2	5 (25)
(2,4)	7 (35)
>4	8 (40)
**LVSI (%)**	
No	7 (35)
Yes	13 (65)
**DSI (%)**	
Negative	2 (10)
<2/3	4 (20)
≥2/3	14 (70)

Abbreviation: SCC, squamous cervical cancer; AC, adenocarcinoma; AS, adenosquamous carcinoma; NEC, neuroendocrine carcinomas.

**Table 2:** Comparison of clinicopathological characteristics in 14 AS patients with different silva pattern.

**Characteristics**	**Pattern A**	**Pattern B**	**Pattern C**	**Gastric type**
**Age**				
Mean ± SD	52.5±5	43.7±10.6	48±11	48±12.8
**FIGO stage (%)**				
1B	2 (100)	2 (66.7)	6 (100)	3 (100)
2A	0 (0)	1 (33.3)	0 (0)	0 (0)
**Adjuvant treatment (%)**				
No	2 (100)	1 (33.3)	1 (16.7)	1 (33.3)
Yes	0 (0)	2 (66.7)	5 (83.3)	2 (66.7)
**HPV infection (%)**				
No	0 (0)	0 (0)	0 (0)	1 (33.3)
Yes	2 (100)	3 (100)	4 (66.7)	0 (0)
Unknown	0 (0)	0 (0)	2 (33.3)	2 (66.7)
**LN metastasis (%)**				
No	2 (100)	2 (66.7)	2 (33.3)	1 (33.3)
Yes	0 (0)	1 (33.3)	4 (66.7)	2 (66.7)
**Surgical margin (%)**				
No	2 (100)	3 (100)	6 (100)	3 (100)
Yes	0 (0)	0 (0)	0 (0)	0 (0)
**Parametrial invasion (%)**				
No	2 (100)	3 (100)	5 (83.3)	2 (66.7)
Yes	0 (0)	0 (0)	1 (16.7)	1 (33.3)
**Tumor size, cm (%)**				
≤2	2 (100)	0 (0)	0 (0)	0 (0)
(2,4)	0 (0)	1 (33.3)	1 (16.7)	2 (66.7)
>4	0 (0)	2 (66.7)	5 (83.3)	1 (33.3)
**LVSI (%)**				
No	1 (50)	2 (66.7)	2 (33.3)	0 (0)
Yes	1 (50)	1 (33.3)	4 (66.7)	3 (100)
**DSI (%)**				
<1/3	2 (0)	0 (0)	0 (0)	2 (14.3)
[1/3-2/3]	0 (0)	1 (33.3)	0 (0)	0 (0)
≥2/3	0 (0)	2 (66.7)	6 (100)	3 (100)

### Small RNA profiles in different subtypes of EAC

**Figure 1:**
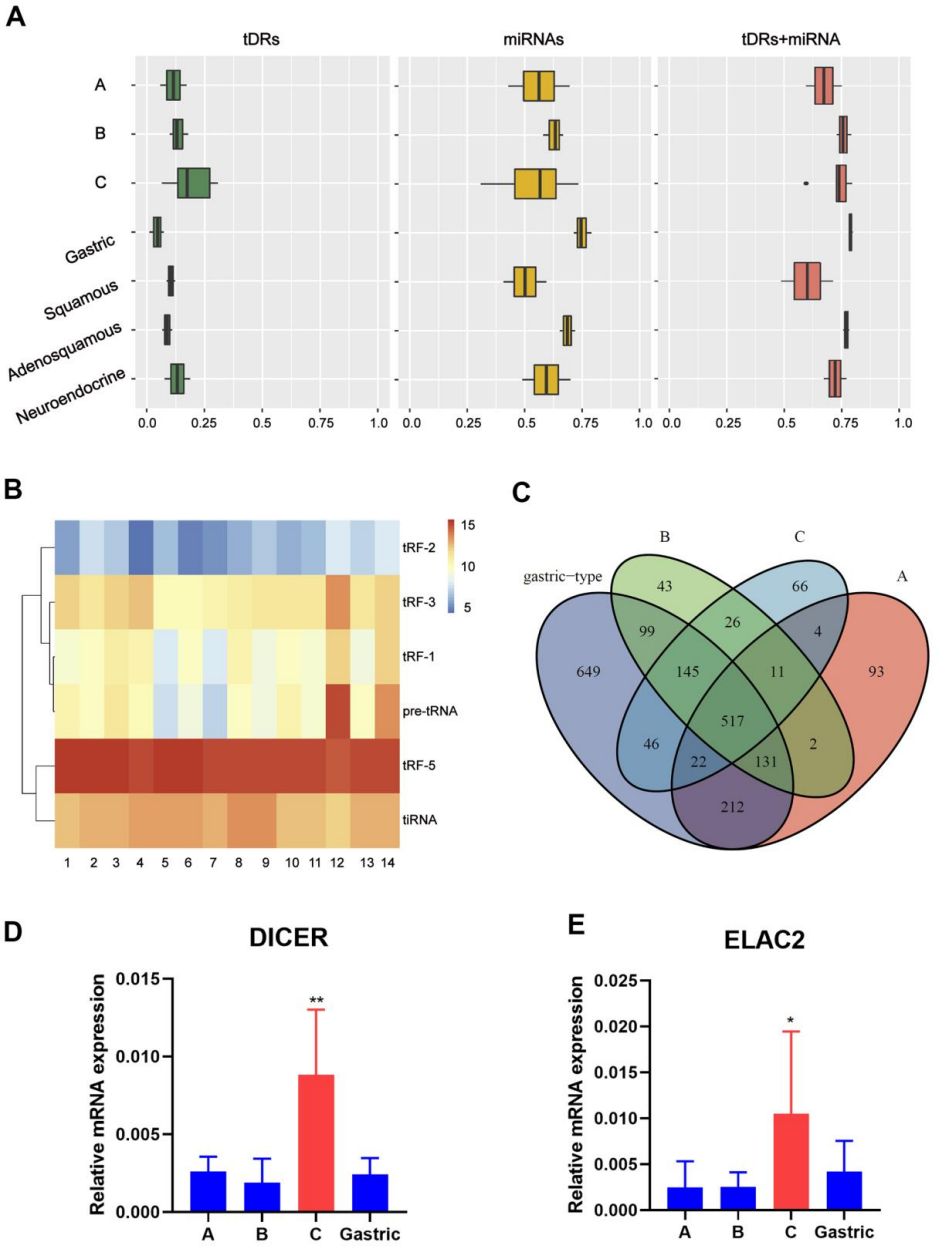
**Catalog of small RNA profiles in different subtypes of EAC.** (**A**) Distribution of RNA Biotypes Differs between Biofluids. Reads mapping to miRNAs, tRNAs or other RNA biotypes as a fraction of total reads mapping to the human transcriptome. Boxes represent median and interquartile ranges, whiskers represent 1.5 times the interquartile range, and dots represent outliers. (**B**) Heatmap depicting all subtypes of tDRs of each sample. (**C**) Venn plot shows that EAC of A, B, C and Gastric type have great differences in the distribution of tDRs types based on the anticodon of amino acids. (**D**, **E**) RT-PCR analysis shows a significant increase of Dicer1 and ELAC2 expression in pattern C compared with pattern A/B/Gastric. (Student t test, *P <0.05).

First of all, the small RNA profiles of the 14 EAC samples with different histological subtypes were shown in Figure 1A. In EAC samples, miRNAs and tDRs accounted for about 75% of all mapped reads. Through literature review, we found that this phenomenon not only exists in EAC, but also in other different types of human samples [19]. In order to further confirm this phenomenon, we performed small RNA sequencing on another six squamous and adeno-squamous cervical cancer samples. There was no significant difference in the fraction of transcriptome-aligned reads for different histologic subtypes of cervical cancer and the total number of tDRs and miRNAs remained relatively balanced. To analyze tDRs reads in further detail, we grouped tDRs based on their biogenesis and relative length. In all subtypes of tDRs, tRF-5 was predominant in abundance (Figure 1B). The Venn diagram shows that EAC of Silva pattern A, B, C and Gastric subtype have great differences in the distribution of tDRs types based on the anticodon of amino acids (Figure 1C). Since tDRs are produced by endonuclease RNase Z, Dicer 1 and ANG, we next compared the expression of these genes between different subtypes of EAC. As shown in Figure 1D, 1E, the expression of Dicer1 and ELAC2 in pattern C was significantly higher than that of other subtypes (Pv0.05, Student T test). Collectively, we discovered that differentially expressed miRNAs and tDRs existed in different histological types of EAC indicating potential clinical value.

### Construction of weighted gene co-expression network by analyzing of miRNA or tDRs

**Figure 2:**
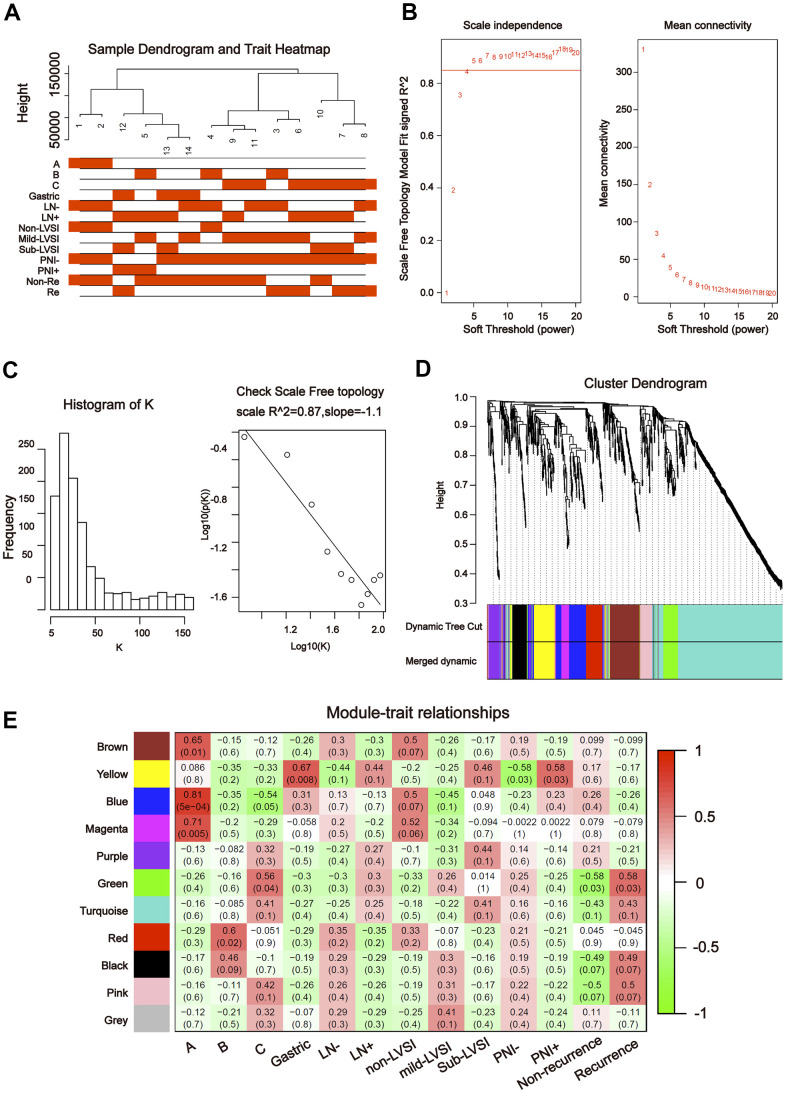
**Construction of weighted gene co-expression network by analyzing of miRNA.** (**A**) Sample dendrogram and clinical variable heatmap based on miRNA. (**B**) Determination of network topology from different soft-threshold powers. (**C**) Check scale-free topology. (**D**) Clustering dendrograms of identified co-expressed miRNAs in modules. (**E**) Heatmaps of the correlation between eigengene and clinical traits based on miRNA.

**Figure 3:**
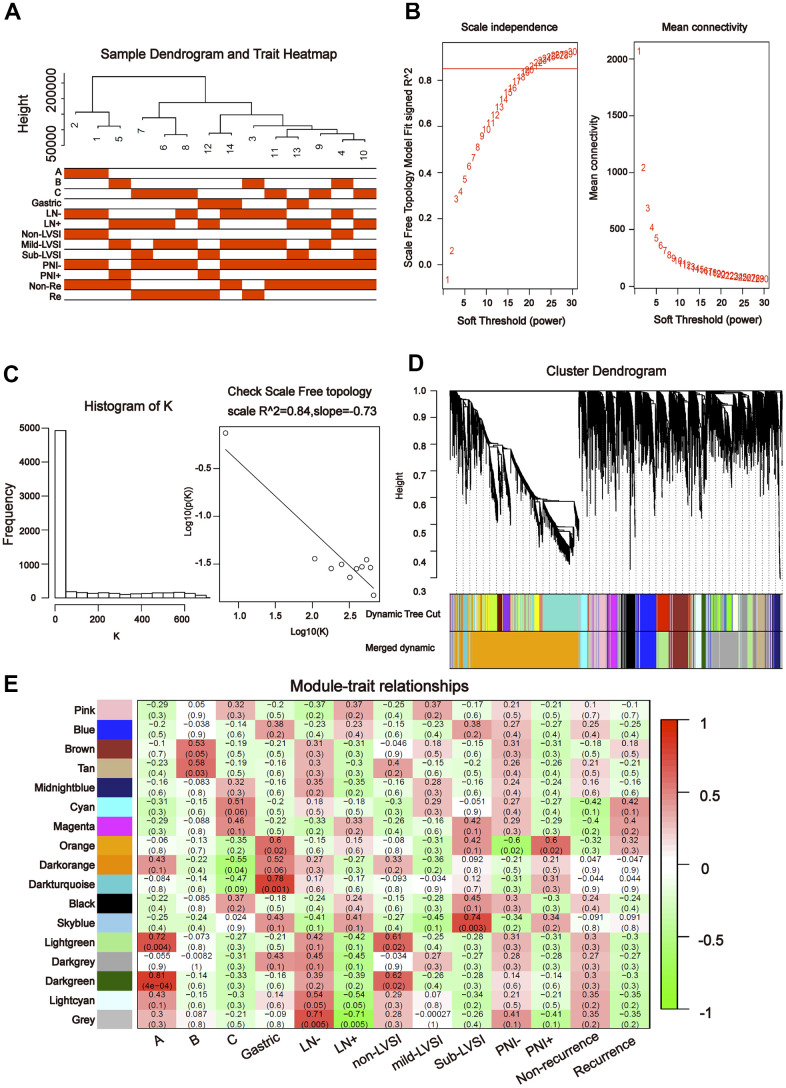
**Construction of weighted gene co-expression network by analyzing of tDRs.** (**A**) Sample dendrogram and clinical variable heatmap based on tDRs. (**B**) Determination of network topology from different soft-threshold powers. (**C**) Check scale-free topology. (**D**) Clustering dendrograms of identified co-expressed tDRs in modules. (**E**) Heatmaps of the correlation between eigengene and clinical traits based on tDRs.

To further explore the association between the differentially expressed miRNAs and tDRs and clinical traits, we conducted weighted gene co-expression network analysis (WGCNA). The investigated clinical phenotypes included Silva pattern A/B/C/Gastric subtypes, non-LNM (lymph node metastasis)/LNM, non-LVSI/mild-LVSI/Substantial-LVSI, non-PNI (perineural invasion) /PNI and non-recurrence/recurrence (Figure 2A). By setting soft-thresholding power as 4 (scale free *R*^2^ = 0.87) and cut height as 0.25, we identified 10 modules (Figure 2B–2D; non-clustering miRNA/tDRs shown in grey) in the miRNA profile. As shown in Figure 2E, some modules correlated significantly (*P* < 0.01) with some of the indicated variables. The blue module was positively correlated with Silva pattern A (*R* =0.81, *P* < 0.001) and the yellow module was positively correlated with Gastric subtype (*R* =0.67, *P* < 0.01). By setting soft-thresholding power as 20 (scale free *R*^2^ = 0.84) and cut height as 0.25, we identified 16 modules (Figure 3A–3D; non-clustering DEGs shown in grey) in the tDRs profile. The dark green module was positively correlated with the Silva pattern A (*R* =0.81, *P* < 0.001) while the dark turquoise module was significantly associated with the Gastric subtype (*R* =0.78, *P* < 0.005). Moreover, the sky blue module was positively correlated with the substantial-LVSI (*R* =0.81, *P* < 0.001) (Figure 3E).

### Screening for hub miRNA/tDRs and functional enrichment analysis of hub miRNA/ tDRs target genes

**Figure 4:**
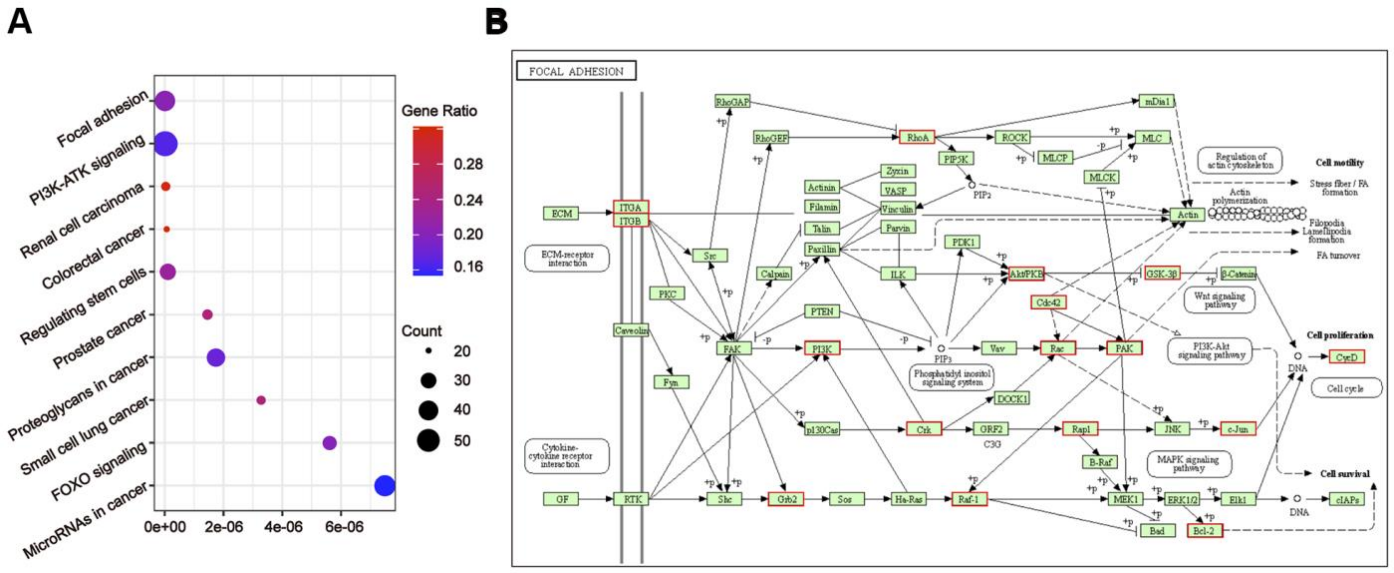
**Gene ontology enrichment analyses with the target genes of hub miRNA/tDRs in silva pattern A.** (**A**) KEGG pathway analysis for the target genes of hsa-miR-101-3p, hsa-miR-195-5p, tRF-1:32-Val-CAC-3 and tRF-1:28-Gly-CCC-2. (**B**) Mapping of focal adhesion signaling pathway. Red marked nodes are associated with hub miRNA/tDRs.

**Figure 5:**
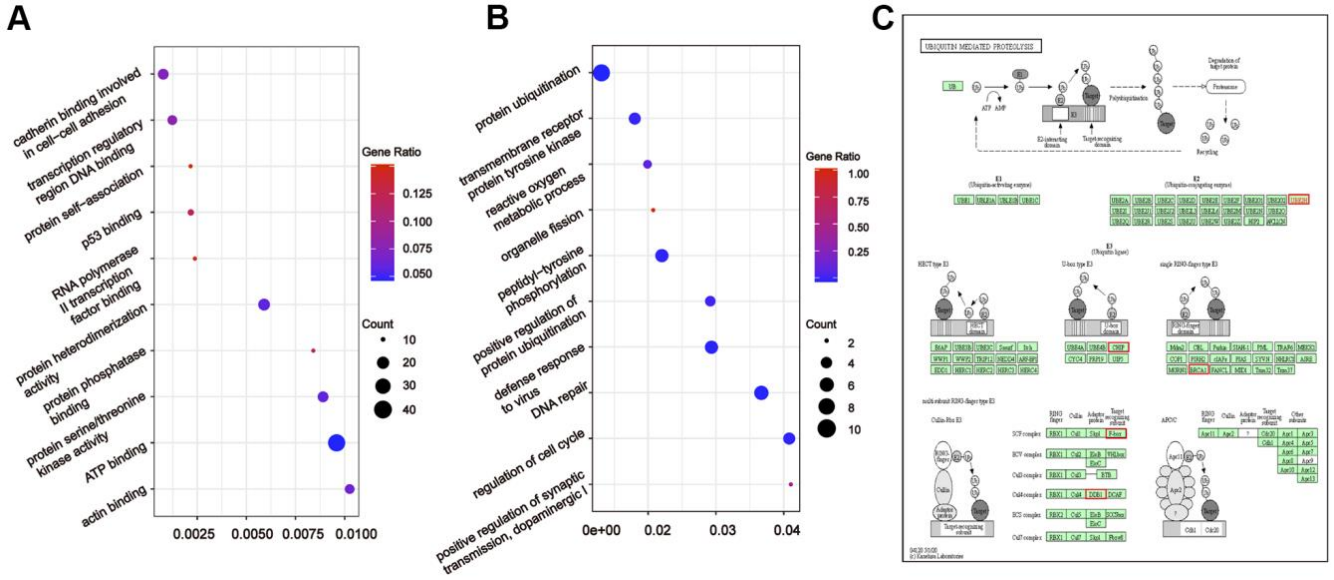
**Gene ontology enrichment analyses with the target genes of hub miRNA/tDRs in silva pattern gastric and substantial-LVSI.** (**A**) KEGG pathway analysis for the target genes of hsa-miR-214-3p, Other-13:26-tRNA-Lys-CTT-1-M11 and Other-2:23-tRNA-Val-AAC-1-M7 in Silva pattern Gastric. (**B**) KEGG pathway analysis for the target genes of other-14:33-tRNA-Lys-CTT-1-M2, other-3:35-tRNA-Lys-CTT-1-M2 and other-14:28-tRNA-Lys-CTT-10 in substantial-LVSI patients. (**C**) Mapping of ubiquitin mediated proteolysis pathway. Red marked nodes are associated with hub miRNA/tDRs.

To further understand the relationship between modules and clinical phenotypes, we screened for the hub miRNAs or tDRs in specific modules. The cut-off values of miRNA/tDRs were: module membership (MM) >0.8, correlation coefficient of clinical trait > 0.7, and the counts of per million mapped reads (CPM) > 100. Notably, four miRNA/tDRs were identified from the miRNAs and tDRs profiles in the miRNA-based blue coexpression module and tDRs-based dark green coexpression module to be positively associated with the Silva pattern A, including hsa-miR-101-3p, hsa-miR-195-5p, tRF-1:32-Val-CAC-3 and tRF-1:28-Gly-CCC-2. To demonstrate potential biological functions of the hub miRNA/tDRs for Silva pattern A, we performed GO and KEGG analyses with the target genes of the miRNA/tDRs and concluded that the most enriched signal pathways for the Silva pattern A were focal adhesion and PI3K-Akt-mTOR signaling pathways which were common tumorigenesis-related pathways (Figure 4A, 4B). In addition, three miRNA/tDRs were identified positively associated with the Gastric subtype, including hsa-miR-214-3p, Other-13:26-tRNA-Lys-CTT-1-M11 and Other-2:23-tRNA-Val-AAC-1-M7. GO and KEGG analysis showed that cadherin binding involved in cell-cell adhesion was the most enriched signal pathway for Gastric subtype, potentially implying a unique cell-cell interaction (Figure 5A). Furthermore, a total of 3 hub tRNAs were discovered positively associated with substantial-LVSI, including other-14:33-tRNA-Lys-CTT-1-M2, other-3:35-tRNA-Lys-CTT-1-M2 and other-14:28-tRNA-Lys-CTT-10. GO and KEGG analysis suggested that the most relevant pathway was ubiquitin mediated proteolysis pathway (Figure 5B, 5C). To summarize, we identified several hub miRNA/tDRs closely associated with significant clinical phenotypes such as Silva pattern A, gastric subtype and substantial LVSI exhibiting concordant biological functions.

### qRT-PCR confirmed the hub miRNA/tDRs

**Figure 6:**
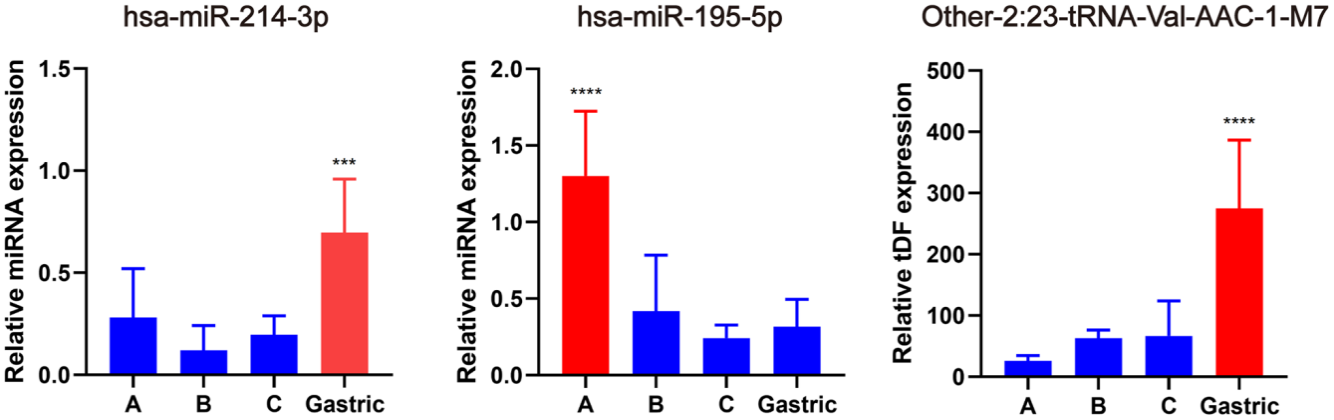
**Validation of hub miRNA/tDRs in different subtypes of EAC tissues.** A, B and C represents Silva A, B, C pattern respectively. All data were analyzed using Student’s t-test. The asterisk indicates a significant difference between two groups (***P< 0.001, ****P< 0.0001).

Subsequently, qRT-PCR was used to validate the expression of the hub miRNAs/tDRs that were identified from WGCNA analysis in EAC samples, including seven tDRs and two miRNAs. Among them, the expression of the following four tRNAs (other-14:33-tRNA-Lys-CTT-1-M2, tRF-1:32-Val-CAC-3, tRF-1:28-Gly-CCC-2, and other-14:28-tRNA-Lys-CTT-10) were too low to be quantized with qPCR. No significant differences were found for other-3:35-tRNA-Lys-CTT-1-M2 and Other-13:26-tRNA-Lys-CTT-1-M11 in different subtypes of EAC tissues (*P* > 0.05), whereas significant differences were verified for hsa-miR-214-3p, hsa-miR-195-5p and Other-2:23-tRNA-Val-AAC-1-M7 (Figure 6) indicating promising value to become potential biomarkers for EAC.

## DISCUSSION

Cervical carcinoma is one of the most common cancers of women, which has a higher incidence in developing countries. With the popularization of the screening programs, the incidence of cervical carcinoma, especially squamous cell carcinoma has declined. However, because human papilloma virus (HPV) infection does not always accompany cervical adenocarcinoma and the lesion can be endogenous, the existing screening methods are less effective in the diagnosis of cervical adenocarcinoma. Compared with cervical squamous cell carcinoma, the prognosis for cervical adenocarcinoma is poor. Small non-coding RNA is an important part of epigenetic modification, which can modify gene expression without changing DNA sequence and plays a fundamental role in diverse biologic processes [20]. Deregulation of miRNAs, piRNAs and tDRs, e.g., has been implicated in several metabolic diseases as well as in cancer [21–23].

In this study, we first performed a standardized RNA-sequencing method to explore small RNAs in different subtypes of EAC. This work expands researchers' understanding of the transcriptome of cervical cancer and fills the gap of small RNAs in EAC research. In each EAC subtype tested, the total number of miRNAs and tDRs maintained a relative balance, at about 75%. These results were also verified in another 6 non-EAC cervical cancer samples. Through literature review, we found that this phenomenon not only exists in cervical cancer, but also in other different types of samples: the total amount of miRNAs and tDRs reached a relative balance in different kinds of human biofluids [19]. Our findings demonstrate the dynamic balance between miRNAs and tDRs, which might lay a foundation for further study of the mechanism.

Compared with other bioinformatics methods, WGCNA focuses on the association between clinical traits and co-expression modules leading to higher biological significance and reliability [24]. To explore the correlations between small RNAs expression and clinical characteristics of EAC, we performed WGCNA analysis and screened for hub small RNAs. Further target gene prediction and gene ontology analysis demonstrated that the most relevant signal pathways for the Silva pattern A of EAC were PI3K-Akt-mTOR signaling pathways and focal adhesion, which is consistent with the clinicopathological characteristics of the Silva pattern A. It is well known that PI3K-Akt-mTOR signaling pathway regulates fundamental cellular functions such as transcription, proliferation and metabolism, component genes of which are commonly activated in cancer [25]. Additionally, the dynamic status of focal adhesion was closely associated with cell migration. Silva pattern A EAC is featured by an obvious boundary from the surrounding stroma without invasiveness, which might be attributed to focal adhesion. In contrast, hub small RNAs related with Gastric subtype of EAC were enriched in cadherin binding involved in cell-cell adhesion. Dysfunction of cadherin adhesion is closely related to the epithelial transition in cancer cells and tumor invasiveness [26], in accordance with the great malignancy and strong invasiveness of the Gastric subtype. Moreover, we discovered that the signal pathways related with substantial LVSI was ubiquitin mediated proteolysis pathway. To further verify this correlation, we extracted DEGs from patients with positive and negative LVSI in the Cancer Genome Atlas (TCGA) cervical cancer cohort and found that the DEGs were also enriched in proteolysis pathway. These results confirmed a close association between proteolysis signaling pathway and the clinical phenotype of LVSI, providing a research direction for subsequent functional experiments.

In conclusion, we constructed an extensive catalog of small RNAs in EAC tissues through small RNA sequencing approaches. This work not only provides a resource for investigators exploring the distribution and function of small RNAs but also helps to identify small RNAs that may have potential as novel biomarkers for EAC.

## MATERIALS AND METHODS

### Ethical approval

The researchers were granted approval to conduct the research by Departmental Research Ethics Committee at Obstetrics and Gynecology Hospital, Fudan University, Shanghai, China. The study protocol was approved by the institutional review board of Obstetrics and Gynecology Hospital. All participants signed informed consent forms.

### RNA extraction

According to the manufacturer's instructions, TRIzol (Invitrogen, CA, USA) was used to extract total RNA from frozen tissue. Use NanoDrop ND-1000 instrument (NanoDrop-Thermo, DE, USA) to evaluate the quantity and concentration of each RNA sample, and check the integrity by agarose gel electrophoresis.

### Library preparation and sequencing

Before preparing the total RNA sample library, perform the following treatments to remove the modification of RNA interference small RNA sequence library construction: 3-aminoacyl (charged) deacylation to 3-OH for 3-adapter connection; 3-cP (2,3-cyclic phosphoric acid) was removed to 3-OH for 3-adapter connection; 5-OH (hydroxyl group) was phosphorylated to 5-P for 5-Adapter connection; m1A and m3C are removed for effective reverse transcription. The size of the sequencing library is selected for the RNA biotype, and the sequencing gel cutter was performed with an automatic sequencer, including 3 adaptor and 5'- adaptor connection, cDNA synthesis and library PCR amplification. The quality of the library was checked using Agilent Bioanalyzer 2100 (Invitrogen, CA, USA). Sequencing was performed on Illumina NextSeq 500 (Illumina, DE, USA) by Aksomics (Shanghai, China).

### Sequencing data analysis

Solexa pipeline was used to perform image analysis and base calling. Sequencing quality was examined by FastQC software and trimmed reads were aligned to mature-tRNA and pre-tRNA sequence getting from the Genomic tRNA Database using NovoAlign software. The remaining reads were aligned to transcriptome sequences (mRNA/rRNA/snRNA/snoRNA/piRNA/miRNA). The processed data has been uploaded to the Supplementary Material.

### Weighted gene co-expression network analysis (WGCNA)

WGCNA was applied to the count per million (CPM) expression data. In the WGCNA model based on miRNA data, when β = 4, the scale R2 was 0.88, which could obtain higher average connectivity degree. In the WGCNA model based on tDRs data, when β = 20, the scale R2 was 0.84, which could obtain higher average connectivity degree. In the cluster dendrogram, cluster highly absolutely related genes into the first group of modules (Dynamic Tree Cut), and then merge related modules (r>0.80) together (Merged dynamic).

### Gene ontology analysis

Target gene prediction of tDRs was conducted based on TargetScan and Miranda algorithms (Supplementary Table 1). Putative genes with context less than -0.3 were selected to performed Gene Ontology (GO)analysis. miRTarBase database was used to predict target genes of miRNA. Putative genes with experimental verification data were selected to perform Gene Ontology (GO) analysis.

### qRT-PCR validation

The hub genes were further confirmed by qPCR. U6 small nuclear RNA (snRNA) was used as inter control. Firstly, total RNA was extracted using TRIzol reagent (Invitrogen). Then, the total RNA was pretreated using rtStar™ tRF and tiRNA Pretreatment Kit (Cat# AS-FS-005, Arraystar) to remove 3’-aminoacyl and 3’-cP for 3’ adaptor ligation, phosphorylates 5’-OH for 5’-adaptor ligation, and demethylates m1A, m1G, and m3C for efficient cDNA reverse transcription. Purify the RNA by phenol-chloroform extraction and ethanol precipitation. The post-treated RNA was reverse-transcribed into cDNA using rtStar™ First-Strand cDNA Synthesis Kit (3’ and 5’ adaptor) (Cat# AS-FS-003, Arraystar) according to the manufacturer’s protocols. Then, qPCR amplification was performed using ViiA 7 Real-time PCR System (Applied Biosystems, MA, USA). The standard curves method was used for analysis of the differential expression of tDRs in samples. For each tDRs or U6 that needs to be measured, select a cDNA template that is sure to express the tDRs or U6 for qPCR amplification. Perform a 10-fold gradient dilution of the qPCR product: set the qPCR product concentration to 1, respectively dilute to 1 × 10^-1^, 1 × 10^-2^, 1 × 10^-3^, 1 × 10^-4^, 1 × 10^-5^,1×10^-6^, 1×10^-7^ these are several gradient concentrations of cDNA used as template to produce standard curves to quantify the efficiency (e) of primers in qPCR. PCR was performed in 10 μl reaction volume, including 2 μl of cDNA, 5 μl of 2×master mix, 0.5 μl of forward primer (10 μM), 0.5 μl of reverse primer (10 μM) and 2 μl of double-distilled water. The reaction was pre-denatured at 95° C for 10 min, followed by 40 amplification cycles at 95° C for 10 s, 60° C for 60 s. According to the gradient dilution cDNA standard curve, the relative tDRs expression levels of each samples are directly generated by the machine and were normalized by U6. All reactions were performed in triplicate. The primers used in this study were in the Supplementary Table 2.

### Statistical analysis

Categorical variables were described as the frequency (n) and proportion (%) while continuous variables were presented as the mean ± SE (standard error). Differences in the variables between groups were tested using t-tests, nonparametric tests, chi-square tests, or ANOVA tests [27]. All hypothetical tests were two-sided, and a *p* value less than 0.05 was considered statistically significant.

### Data availability statement

The data reported in this paper have been deposited in the Supplementary Material. Sequence data have been deposited in the NCBI Gene Expression Omnibus (GEO: GSE176579).

## Supplementary Material

Supplementary Table 1

Supplementary Table 2
